# Silica nanoparticles alleviate the immunosuppression, oxidative stress, biochemical, behavioral, and histopathological alterations induced by *Aeromonas veronii* infection in African catfish (*Clarias gariepinus*)

**DOI:** 10.1007/s10695-023-01274-6

**Published:** 2023-12-07

**Authors:** Heba H. Mahboub, Wafaa M. Gad, Enas K. Aziz, Mona Abdelghany Nasr, Esraa M. Fahmy, Dina Mohamed Mansour, Nesma Rasheed, Hanaa S. Ali, Sameh H. Ismail, Afaf N. Abdel Rahman

**Affiliations:** 1 Department of Aquatic Animal Medicine, Faculty of Veterinary Medicine, Zagazig University, Box 44511, Sharkia, Zagazig, PO Egypt; 2 Department of Bacteriology, Animal Health Research Institute (AHRI) (Mansoura Branch), Agriculture Research Center (ARC), Box 246 Dokki, Giza, PO 12618 Egypt; 3 Department of Husbandry and Animal Wealth Development, Faculty of Veterinary Medicine, University of Sadat, Box 32897, Menofia, Sadat City, PO Egypt; 4 Department of Anatomy and Embryology, Faculty of Veterinary Medicine, University of Sadat City, Box 32897, Menofia, Sadat City, PO Egypt; 5 Department of Pharmacology, Faculty of Veterinary Medicine, Zagazig University, Box 44511, Sharkia, Zagazig, PO Egypt; 6 Department of Fish Diseases and Management, Animal Health Research Institute (AHRI), Agriculture Research Center (ARC) (Hurghada branch), Box 246 Dokki, Giza, PO 12618 Egypt; 7 Department of Pathology, Animal Health Research Institute (AHRI) (Mansoura Branch), Agriculture Research Center (ARC), Box 246 Dokki, Giza, PO 12618 Egypt; 8 Faculty of Nanotechnology for Postgraduate Studies, Cairo University, Sheikh Zayed Branch Campus, Sheikh Zayed City, Box 12588, Giza, PO Egypt

**Keywords:** *Aeromonas veronii*, Behavior, *Clarias gariepinus*, Immunohistochemistry, Physiology, Silica nanoparticles

## Abstract

In the aquaculture industry, silica nanoparticles (SiNPs) have great significance, mainly for confronting diseases. Therefore, the present study aims to assess the antibacterial efficiency of SiNPs as a versatile trial against *Aeromonas veronii* infection in African catfish (*Clarias gariepinus*). Further, we investigated the influence of SiNPs in palliating the immune-antioxidant stress biochemical, ethological, and histopathological alterations induced by *A. veronii*. The experiment was conducted for 10 days, and about 120 fish were distributed into four groups at random, with 30 fish each. The first group is a control that was neither exposed to infection nor SiNPs. The second group (SiNPs) was vulnerable to SiNPs at a concentration of 20 mg/L in water. The third group was experimentally infected with *A. veronii* at a concentration of 1.5 × 10^7^ CFU/mL. The fourth group (*A. veronii +* SiNPs) was exposed to SiNPs and infected with *A. veronii*. Results outlined that *A. veronii* infection induced behavioral alterations and suppression of immune-antioxidant responses that appeared as a clear decline in protein profile indices, complement 3, lysozyme activity, glutathione peroxidase, and total antioxidant capacity. The kidney and liver function biomarkers (creatinine, urea, alkaline phosphatase, and alanine aminotransferase) and lipid peroxide (malondialdehyde) were substantially increased in the *A. veronii* group, with marked histopathological changes and immunohistochemical alterations in these tissues. Interestingly, the exposure to SiNPs resulted in a clear improvement in all measured biomarkers and a noticeable regeneration of the histopathological changes. Overall, it will establish that SiNPs are a new, successful tool for opposing immunological, antioxidant, physiological, and histopathological alterations induced by *A. veronii* infection.

## Introduction

The aquaculture industry supplies humans with a high source of animal protein, which is rich in nutrients (FAO 2022). Globally, the extensive production of edible fish puts fish under many stressors, particularly bacterial infections (Rajme-Manzur et al. 2021; Irshath et al. 2023). Aeromonas infection is one of the most devastating pathogenic bacteria associated with massive mortalities during fish culture (Soni et al. 2021). *Aeromonas veronii* is a Gram-negative bacterium responsible for huge losses in aquaculture production worldwide (Adhikary et al. 2023). In fish, previous reports address the hazards of *A. veronii*, including the occurrence of ulceration in Chinese long-snout catfish (*Leiocassis longirostris Günther*) (Cai et al. 2012), mass mortalities in dark sleeper (*Odontobutis potamophila*) (Liu et al. 2022), African catfish (*Clarias gariepinus*) (Li et al. 2019), and Nile tilapia (*Oreochromis niloticus*) (Reda et al. 2021).

Recently, extensive investigations have been carried out in the progress of innovative nanomaterials that are environmentally friendly, synthesized at low cost, and highly effective against pathogens (Ismail et al. 2021; Ibrahim et al. 2022; Abdel Rahman et al. 2023b). In comparison with other developed nanomaterials, silica nanoparticles (SiNPs) are characterized by higher stability chemical, mechanical, and thermal (up to 1500 °C) and contain higher contents in hydroxyl-containing groups (Singh et al. 2017). Also, SiNPs have been recorded in the food industry and agriculture owing to their safety application plus their promising effect on the development and growth of plants, particularly under stress conditions (Winkler et al. 2016; Bhat et al. 2021). They have been proven to have potent antibacterial activity as they are characterized by perfect synergic activity depending on reactive oxygen species (ROS) to destroy bacteria because of penetrating the microbial membrane (Karaman et al. 2018; Bernardos et al. 2019; Tabriz et al. 2023). In the aquaculture sector, SiNPs have a major verified role in fostering fish immunity and antioxidant response against heavy metal toxicity and thus improve the health of *C. gariepinus* (Mahboub et al. 2022b). More recently, SiNPs have been reported to stimulate immune parameters and enhance gene expression, and accordingly, antagonize the immune dysfunction and gene down-regulation elicited by *A. veronii* bacterial infection in *C. gariepinus* (Abdel Rahman et al. 2023a). Supplementing *O. niloticus* in SiNP-enriched diets boosts the ionic exchange mechanism and enhances growth efficacy and hematological picture indicated by augmenting growth indices and blood biomarkers (Alandiyjany et al. 2021; Bashar et al. 2021).

Hence, few studies assess the antibacterial effect of SiNPs. Therefore, the current perspective investigates the efficacy of the aqueous addition of SiNPs against *A. veronii* infection plus studying their pivotal role against immune-antioxidant suppression, hepatic-renal dysfunction, histopathological, and immunohistochemical alterations produced by *A. veronii* challenge in *C. gariepinus*.

## Materials and methods

### Ethical acceptance and bacterial strain (*A*. *veronii*)

By the approval number (ZU-IACUC/2/F/309/2022), the Ethical Committee for the Used Animals authorized the ongoing research at Zagazig University in Egypt. At the Aquatic Animal Medicine Department of the Faculty of Veterinary Medicine, Zagazig University, Egypt, *A. veronii* was recovered from naturally infected African catfish. Additionally, its pathogenicity was confirmed. *A. veronii* was cultivated for a day at 26 °C on tryptic soy agar (TSA) of HiMedia®. One colony was picked to incubate for an additional day at 26 °C in tryptic soy broth (TSB) of HiMedia®. The pellet from the *A. veronii* cultured broth was extracted using a centrifuge at 3000 rpm for 10 min, and it was then suspended in a sterile phosphate-buffered saline (PBS). The lethal dose (LD_50_) was previously determined by Abdel Rahman et al. (2023a), which was 8.7 × 10^8^ CFU/mL, and 1.5 × 10^7^ CFU/mL was employed as a sub-lethal dosage in the treatment assay.

### Fish rearing and experimental design

Two hundred and twenty African catfish (90 ± 6.19 g) were purchased from a private fish farm (Al-Abbassa) in Sharkia Governorate, Egypt. For acclimatization, the fish were kept for 14 days in 100-L well-aerated aquaria (10 fish/aquarium), whereas the water was partially exchanged (25%). The fish were fed a commercial diet at a percentage of 3% of their body weight. Temperature, dissolved oxygen, pH, and ammonia of the acclimating water were all monitored every day during the acclimation and trial, and they recorded 25 ± 1.5 °C, 6.4 ± 0.6 mg/L, 6.3 ± 0.2, and 0.01 ± 0.02 mg/L respectively.

For 10 days (treatment trial), about 120 fish were divided into four groups in random with 30 fish each (3 replicate/group; 10 fish/replicate). The first group (control) was neither exposed to infection nor SiNPs. The second group (SiNPs) was exposed to SiNPs at a concentration of 20 mg/L in water (Abdel Rahman et al. 2023a). The third group (*A. veronii*) was experimentally intraperitoneally injected with 0.2 mL of *A. veronii* bacterial suspension at a concentration of 1.5 × 10^7^ CFU/mL (Li et al. 2019). The fourth group (*A. veronii +* SiNPs) was challenged with *A. veronii* and exposed to SiNPs (the same doses as the second and third groups). The SiNPs were synthesized and characterized in a recent publication (Abdel Rahman et al. 2023a). SiNPs were introduced to the aquarium water on the second day of the trial (after the manifestation of clinical signs) and continued for 10 days. To get rid of waste, siphoning was done daily. To maintain the 20 mg/L SiNP concentration after water renewal (three times weekly), a freshly produced SiNP solution was added.

### Behavioral and gross observations

For 10 days, clinical signs and fish behavior in all experimental groups were recorded in each aquarium twice daily from 09:00 a.m. to 03:00 p.m. using a controlled camera with an adjustable timer according to Altman’s (1974) approach. According to Ismail et al. (2009) technique, the spiral movement, activeness, and laterality were recorded. Spiral movement refers to the fish numbers that swim in the aquarium in a spiral pattern with jerks for 3 min each day. Activeness involved fish remaining motionless in a group at the aquarium’s bottom for 3 min each day. Laterality is measured by the fish numbers that shift to the lateral side at the bottom for 3 min each day.

Meanwhile, loss of equilibrium was recorded according to Calfee et al. (2016), which referred to a fish’s failure to consistently hold itself upright in the water column once daily. According to Abdel Rahman et al. (2022), hiding behavior was observed, referring to the fish numbers that remain hidden in the tank’s corners within 3 min per day.

### Sampling

After the study (10 days), fish were chosen at random (9 fish/group) to drain blood samples. A 100 mg/L of benzocaine solution was used for anesthetizing fish (Neiffer and Stamper 2009), and blood was drawn from the caudal blood vessels using anticoagulant-devoid syringes. To obtain serum samples for biochemical, immunological, and antioxidant/oxidant assays, blood samples were centrifuged at 1750 *× g* for 10 min. Samples from kidney and liver tissue (9 fish/group) were collected for histopathological and immunohistochemical analysis.

### Assessment of biochemical parameters

The serum concentration of creatinine was estimated at a wavelength of 340 nm as described by Bartels et al. (1972) using a spectrophotometric protocol depending on the manual Centromic Gmbit kit (German). The analysis of serum urea (Catalog No. MBS9374784), alkaline phosphatase (ALP) (Catalog No. E-EL-R1109), and alanine aminotransferase (ALT) (Catalog No. MBS038444) of MyBioSource Co., CA, USA, were assayed. Moreover, serum levels of total protein (TP) (Catalog No. MBS9917835) and albumin (ALB) (Catalog No. MBS019237) were investigated. The determination was spectrophotometrically according to the standard method of their specific pamphlets using a spectrophotometer (Lambda EZ201; Perkin Elmer). The total globulin level (GLO) was calculated by subtracting ALB from TP.

### Immunological and antioxidant/oxidant assays

The immune indices, including lysozymes activity (LYZ) and complement-3 (C3), were estimated in the current study. The activity of LYZ was measured using inhibition zone protocol in agarose gel plates according to the method of Lee and Yang (2002) using ELISA Kit (Bio-diagnostics, Egypt). C3 level was evaluated by immuno-turbidimetry using separated Eastbiopharm ELISA kits (Hangzhou Eastbiopharm CO., LTD., Torrance, USA) following the method of Abdollahi et al. (2016).

The total antioxidant capacity (TAC) concentration was estimated spectrophotometrically in serum following the illustrated assay of Benzie and Strain (1996). The glutathione peroxidase (GPx) and malondialdehyde (MDA) levels were measured using Sigma (MAK085) assay kits depending on the assay of Hamed et al. (2004) and Ohkawa et al. (1979).

### Histopathological and immunohistochemical investigations

Sections from kidney and liver tissues were taken from all groups, then exposed to fixation in 10% buffered neutral formalin, dehydration using ascending grades of alcohol, clearance in xylene, and eventually embedded in paraffin. About 5-μm-thick paraffin sections were collected and then stained with hematoxylin and eosin (H&E) and finally examined by AmScope microscope with a digital camera (Irvine, CA, USA) according to the method of Suvarna et al. (2018).

Following the ABC method (Hsu et al. 1981), the immune-histochemical (IHC) identification of B-cell lymphoma 2 (BCL-2) and cysteine-aspartic proteases (caspase-3) proteins was carried out. An HRP/DAB detection IHC kit (ab80436 Abcam, China) was used according to the manufacturer’s approach. The tissue sections (5 μm thick) that had been formalin fixed and paraffin embedded were then dewaxed and rehydrated. The slices were covered with a 3% hydrogen peroxide solution for 10 min to suppress endogenous peroxidase. Sections were boiled in a solution containing 10 mM sodium citrate buffer (pH 6.0) for 25 min in a microwave to retrieve the antigen (ab64236, Abcam, China).

The non-specific binding was prevented for 60 min using 2% bovine serum albumin in PBS. The sections were incubated with 10 μg/mL primary antibodies against caspase-3 (purified rabbit polyclonal anti-caspase-3 antibody at a dilution of 1:750 of Cell Signaling Technology, Danvers, MA, USA; 9661) and BCL-2 (a mouse anti-human BCL-2 antibody at a dilution of 1:50 of DAKO, Glostrup, Denmark; M0887) overnight at 4 °C. The conjugate was then treated with mouse-specific HRP for 15 min at room temperature. Sections from tissues were counterstained with DAB and Mayer’s hematoxylin to verify the occurrence of an immunostain reaction. The sections were negatively controlled by being dipped in PBS to replace the specific antibody. According to Metwally et al. (2018) protocol, the lesion was graded by computing the percentage of positive cells relative to the total number of cells in the image.

### Data analysis

Firstly, the obtained data were checked for normality using Shapiro-Wilk’s test. One-way analysis of variance (ANOVA) was applied to examine the results of ethological, biochemical, and immuno-antioxidant variables using SPSS version 21 of IBM Corp. (Armonk, USA). Duncan’s multiple range tests were used at a significance level of 0.05 to record the variances between means.

## Results

### Behavioral alterations and clinical signs

Table 1 shows the recorded behaviors where no significant changes (*p >* 0.05) were noted in the hiding and activeness between the SiNP group and the control. The spiral movement, loss of equilibrium, and laterality were not recorded in the SiNPs and the control groups. *A. veronii* infection induced a significant appearance and increased these behaviors. Meanwhile, marked minimizing in these alterations was obvious in the *A. veronii +* SiNP group relative to the *A. veronii* group.

**Table 1:** Effect of *A. veronii* infection and/or SiNPs (20 mg/L) exposure on the various behavioral patterns of African catfish for 10 days (*n* = 30/group)

Parameters	Control	SiNPs	*A. veronii*	*A. veronii* + SiNPs	*p-*value
Spiral movement	0.00 ± 0.00^c^	0.00 ± 0.00^c^	1.27 ± 0.05^a^	0.79 ± 0.02^b^	< 0.0001
Loss of equilibrium	0.00 ± 0.00^c^	0.00 ± 0.00^c^	2.43 ± 0.20^a^	0.96 ± 0.03^a^	< 0.0001
Hiding	0.57 ± 0.10^a^	0.56 ± 0.09 ^a^	1.55 ± 0.09^c^	0.93 ± 0.03^b^	0.0001
Activeness	1.04 ± 0.33^c^	1.02 ± 0.01^c^	9.56 ± 0.56^a^	3.73 ± 0.14^b^	< 0.0001
Laterality	0.00 ± 0.00^c^	0.00 ± 0.00^c^	1.77 ± 0.04^a^	0.48 ± 0.06^b^	< 0.0001

Values (mean ±*SE*) in the same row that do not share the same superscripts are significantly different

**Fig. 1:**
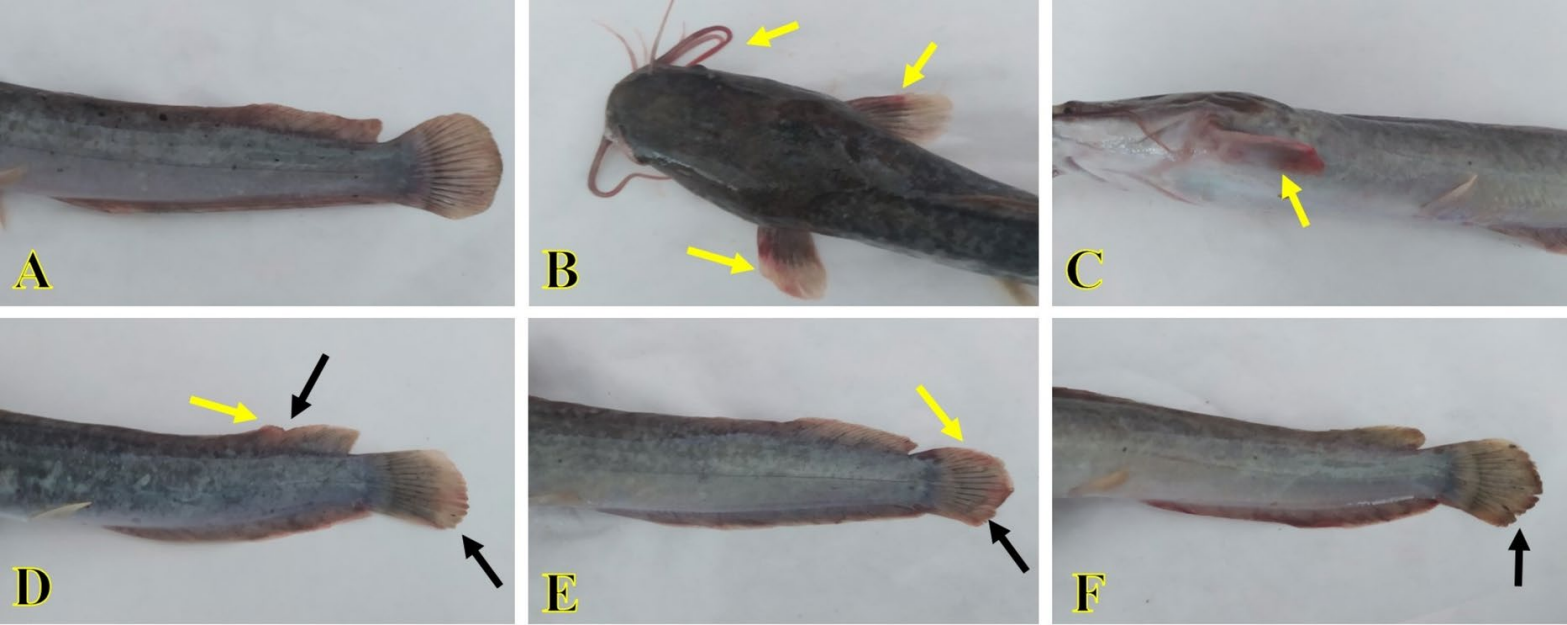
Effect of *A. veronii* infection and/or SiNPs (20 mg/L) exposure on the clinical observation of African catfish for 10 days. **A** Fish of the control or SiNP groups exhibiting no abnormal signs. **B**–**E** The fish of the *A. veronii* group exhibiting dark body coloration, fin rot (dark arrows), and body hemorrhages, especially at the barbles and fins (yellow arrows). **F** Fish of the *A. veronii +* SiNP group exhibiting fin rot at the caudal fin (dark arrow)

No abnormal signs were seen in the control or SiNP group (Fig. 1A). In contrast, several clinical observations in the *A. veronii* group included dark body coloration, excess mucus section, fin rot, and body hemorrhages, especially at the barbles and fins (Fig. 1B–E). SiNP exposure to *A. veronii*-infected fish recovered the prior clinical observations except for moderate fin rot at the caudal fin, which is exhibited in some fish (Fig. 1F).

### Kidney and liver function biomarkers

**Fig. 2:**
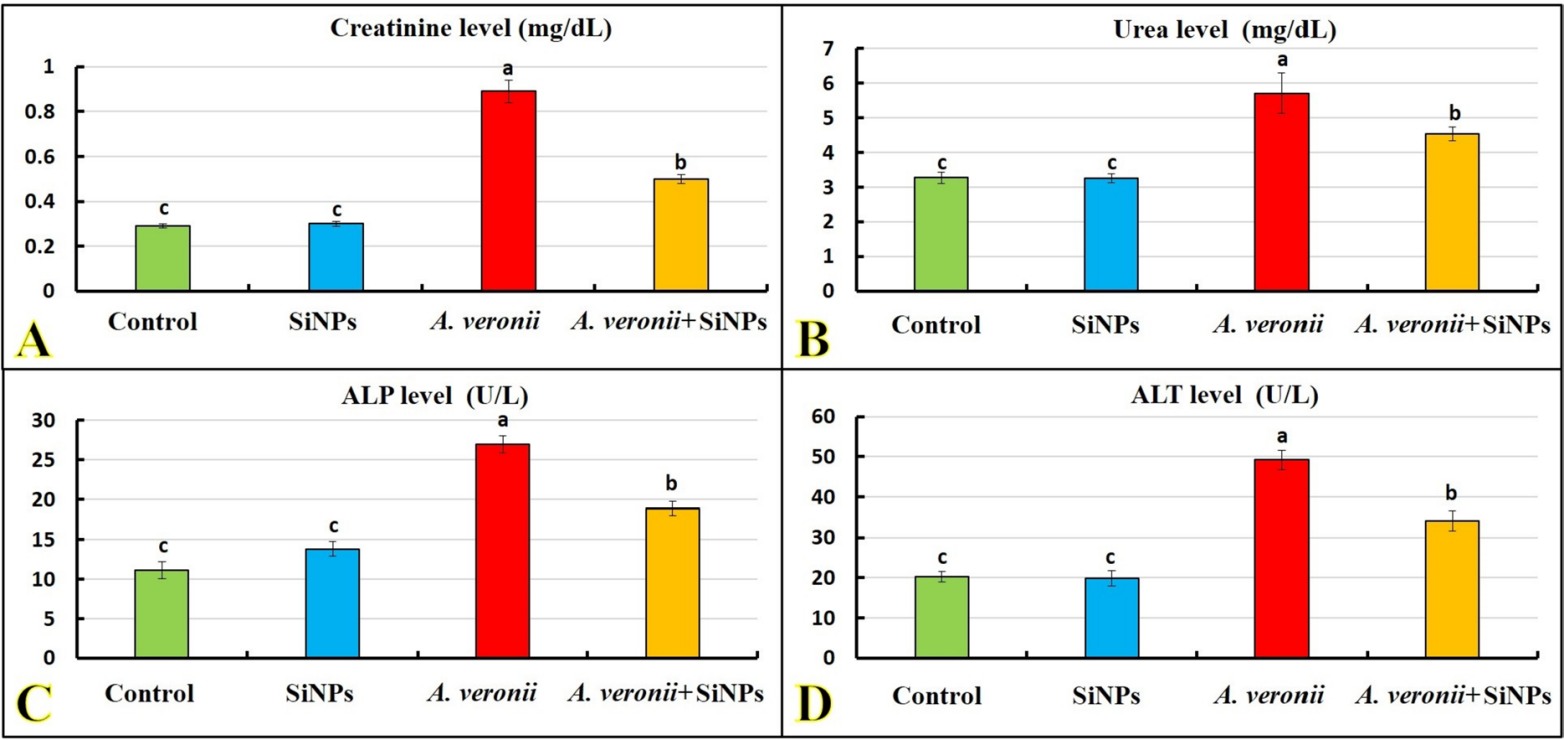
Effect of *A. veronii* infection and/or SiNPs (20 mg/L) exposure on the kidney and liver function biomarkers of African catfish for 10 days (*n* = 9/group). **A** Creatinine level (*p* < 0.0001). **B** Urea level (*p* = 0.002). **C** Alkaline phosphatase level (ALP; *p* < 0.0001). **D** Alanine aminotransferase level (ALT; *p* < 0.0001). Values (mean ± *SE*) that do not share the same superscripts differ significantly

Figure 2A–D shows the serum creatinine, urea, ALP, and ALT results, with a non-significant alteration (*p >* 0.05) between the SiNP group and the control. These variables showed the greatest significant values (*p* < 0.05) in the *A. veronii* group. Contrarily, these biomarkers noticeably decreased in the *A. veronii* + SiNP group compared to the *A. veronii* group.

### Protein profile and immune response

Table 2 reveals that protein profile indices (TP, ALB, and GLO) did not significantly (*p >* 0.05) change in the SiNP group compared to the control; however, the immune response (LYZ and C3) was notably augmented. These biomarkers’ lowest significant (*p* < 0.05) values were obvious in the *A. veronii* group. These biomarkers of the *A. veronii +* SiNP group were substantially enhanced (*p* < 0.05) relative to the *A. veronii* group.

**Table 2:** Effect of *A. veronii* infection and/or SiNPs (20 mg/L) exposure on the protein profile and immune biomarkers of African catfish for 10 days (*n* = 9/group)

Parameters	Control	SiNPs	*A. veronii*	*A. veronii* + SiNPs	*P* value
**Protein profile**					
TP (g/dL)	2.49 ± 0.02^a^	2.48 ± 0.01^a^	2.04 ± 0.03^c^	2.30 ± 0.01^b^	< 0.0001
ALB (g/dL)	1.31 ± 0.03^a^	1.34 ± 0.02^a^	1.08 ± 0.04^b^	1.26 ± 0.02^a^	0.001
GLO (g/dL)	1.18 ± 0.02^a^	1.14 ± 0.01 ^a^	0.96 ± 0.02^c^	1.04 ± 0.01^b^	0.0001
**Immune biomarkers**					
LYZ (ng/mL)	2.50 ± 0.12^b^	4.61 ± 0.16^a^	0.43 ± 0.04^d^	1.79 ± 0.05^c^	< 0.0001
C3 (mg/dL)	27.70 ± 1.19^b^	31.84 ± 0.99 ^a^	12.68 ± 0.56^d^	19.37 ± 0.49^c^	< 0.0001

*TP*, total protein; *ALB*, albumin; *GLO*, globulin; *LYZ*, lysozyme; *C3*, complement3

Values (mean ± *SE*) in the same row that do not share the same superscripts are significantly different

### Antioxidant/oxidant response

**Fig. 3:**
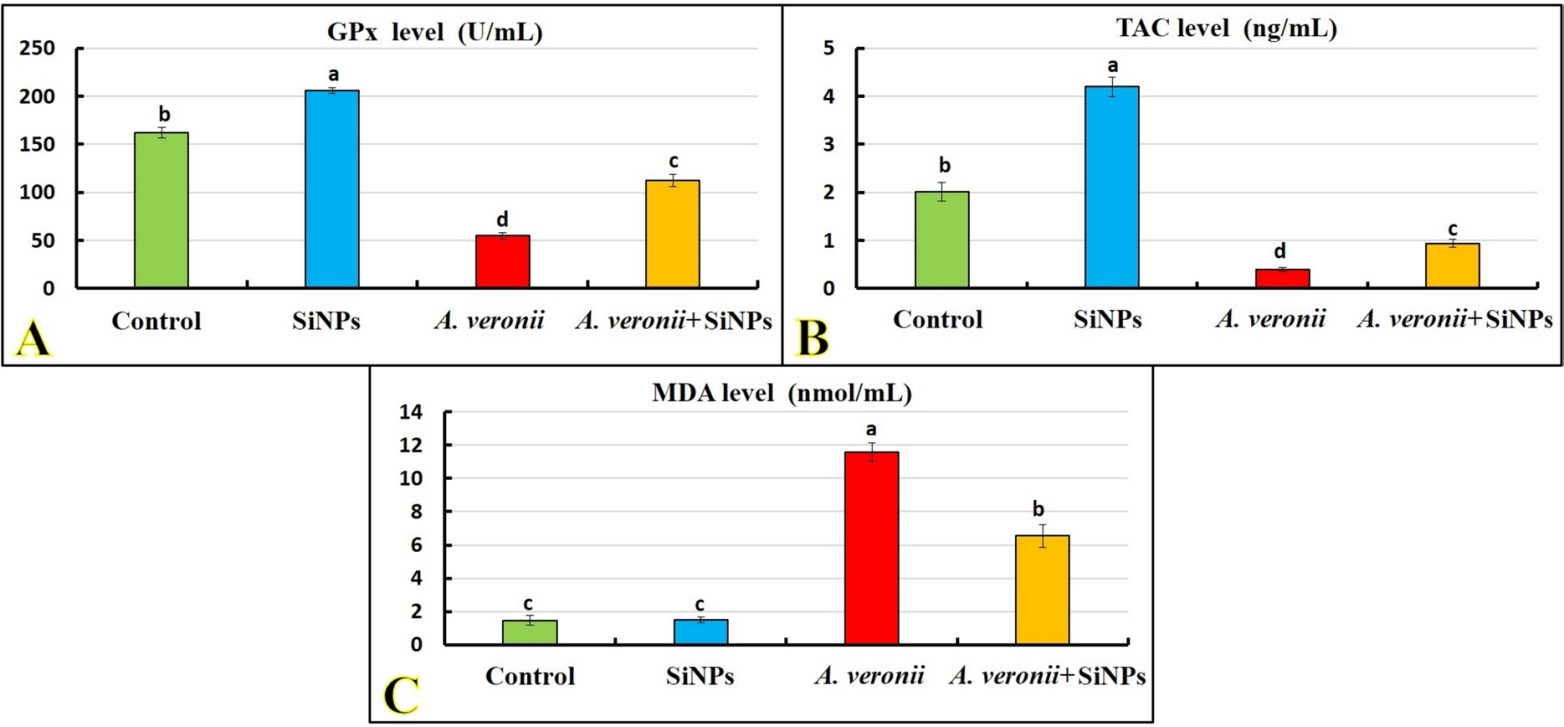
Effect of *A. veronii* infection and/or SiNPs (20 mg/L) exposure on the antioxidant/oxidant biomarkers of African catfish for 10 days (*n* = 9/group). **A** Glutathione peroxidase level (GPx; *p* < 0.0001). **B** Total antioxidant capacity level (TAC; *p* < 0.0001). **C** Malondialdehyde level (MDA; *p* < 0.0001). Values (mean ± *SE*) that do not share the same superscripts differ significantly

Figure 3A–C shows a marked elevation (*p <* 0.05) in GPx and TAC values of the SiNP group compared to the control. Meanwhile, no marked changes were noted for MDA value. *A. veronii* infection induced a noticeable decline (*p <* 0.05) in the GPx and TAC values with an increase of MDA compared to a control group. In contrast, SiNP exposure to *A. veronii-*challenged fish caused a significant augmentation (*p <* 0.05) in GPx and TAC and decreased MDA levels compared to the *A. veronii* group.

### Histopathological findings

**Fig. 4:**
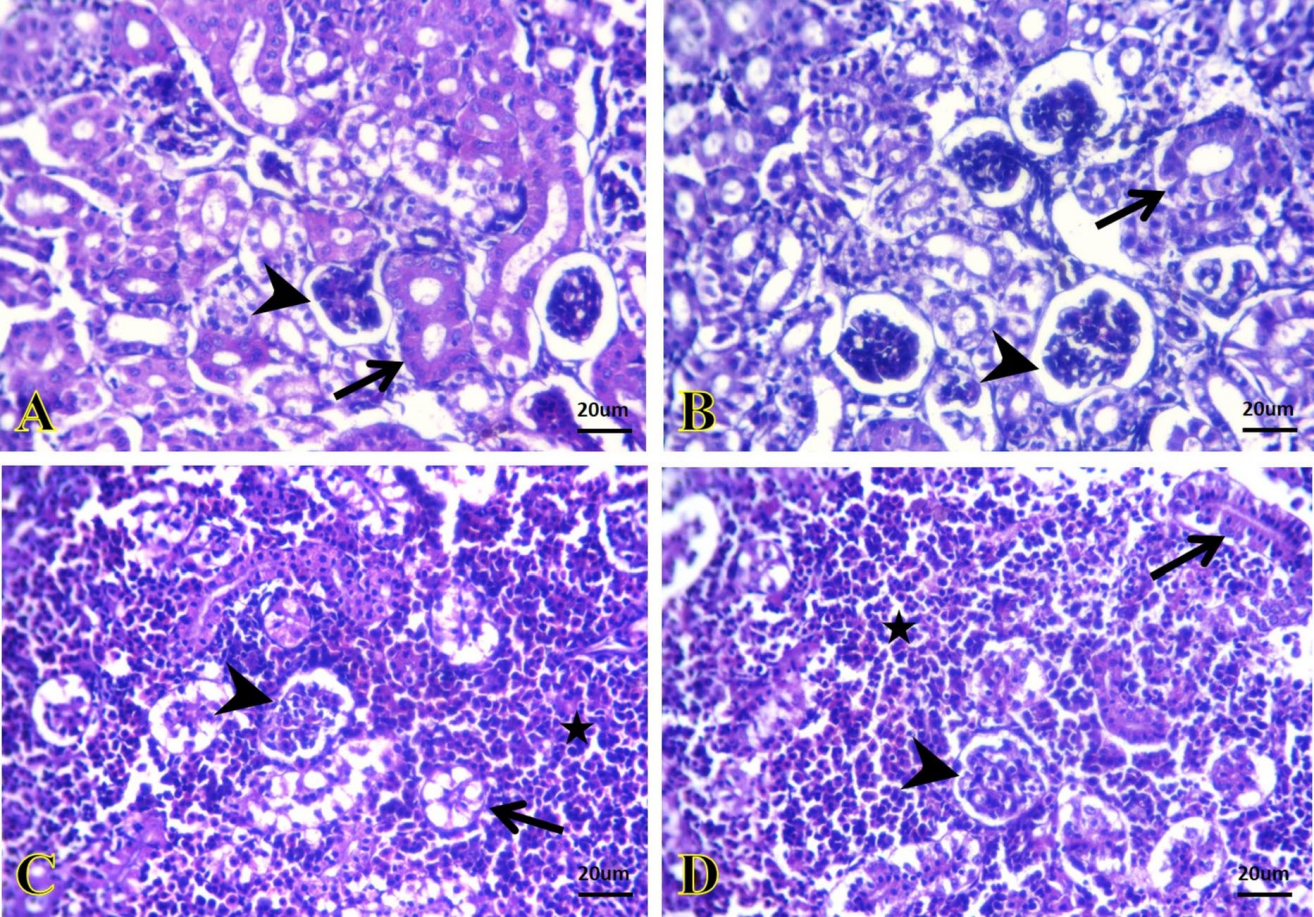
Representative photomicrographs of kidney sections (H&E). **A**, **B** The kidney of the control and SiNPs groups, respectively, exhibiting normal histological structures of glomeruli (arrowheads) and renal tubules (arrows). **C** The kidney of the *A. veronii* group exhibiting hypo-cellular glomerular tufts (arrowhead), oncotic necrosis of a large number of the renal tubular epithelium (arrow), and interstitial infiltration of hemopoietic tissues (star). **D** The kidney of the *A. veronii +* SiNP group exhibiting normal configuration of most glomerular (arrowhead) and tubular structures (arrow) beside the presence of hemopoietic tissues in between (star). Scale bar 20 μm

The kidneys of the control and SiNP groups exhibited normal histological pictures of glomeruli and renal tubules (Fig. 4A and B). *A. veronii* infection induced hypo-cellular glomerular tufts, oncotic necrosis of a large number of renal tubular epithelium, and interstitial infiltration of hemopoietic tissues primarily lymphocytes and erythrocytes (Fig. 4C). On the contrary, kidney of *A. veronii +* SiNP group exhibited normal configuration of most glomerular and tubular structures beside the presence of hemopoietic tissues in between (Fig. 4D).

**Fig. 5:**
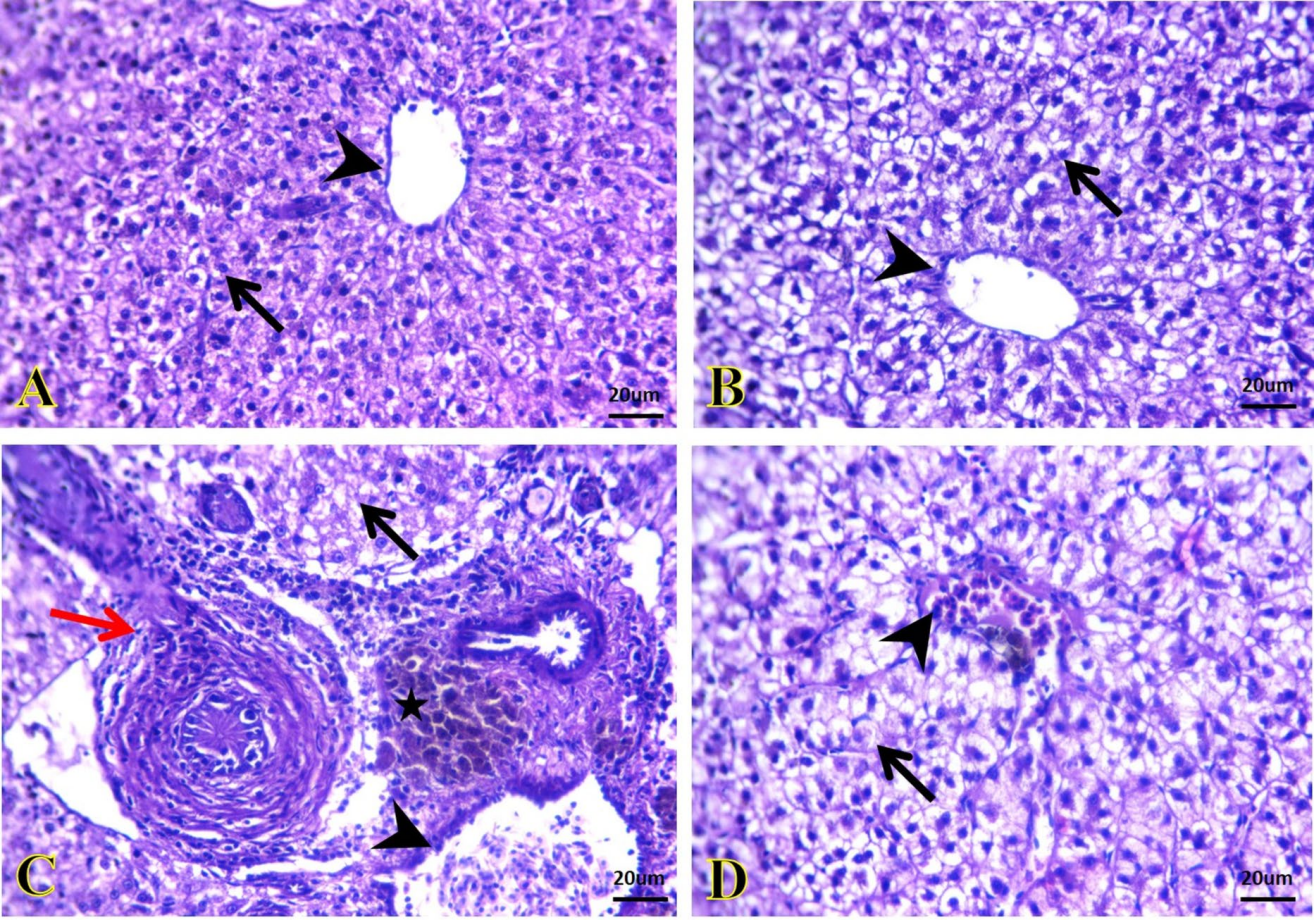
Representative photomicrographs of liver sections (H&E). **A**, **B** The liver of the control and SiNP group, respectively, exhibiting normal histo-architectures of hepatocytes (arrows), sinusoids, and central vein (arrowheads). **C** The liver of the *A. veronii* group exhibiting degenerative changes of the most hepatic parenchyma, few necrotic cells (arrow), congested portal vein (arrowhead), moderate fibrosis, and inflammatory cells infiltrates within the bile duct wall (red arrow) beside the presence of melanomacrophage centers (star). **D** The liver of the *A. veronii +* SiNP group exhibiting mildly congested central vein (arrowhead) and prominent vacuolization (arrow). Scale bar 20 μm

The liver of the control and SiNP groups exhibited normal histo-architectures of hepatocytes, sinusoids, central veins, and portal areas (Fig. 5A and B). In contrast, the liver of the *A. veronii* group exhibited degenerative changes of the most hepatic parenchyma, few necrotic cells, congested portal vein, and chronic cholangitis. Additionally, moderate fibrosis and inflammatory cells infiltrate within the portal area beside the presence of melanomacrophage centers were also seen (Fig. 5C). These alterations were found to be lessened in the *A. veronii +* SiNP group, where mildly congested central vein and prominent vacuolization were frequently seen (Fig. 5D).

### Immunohistochemical findings

**Fig. 6:**
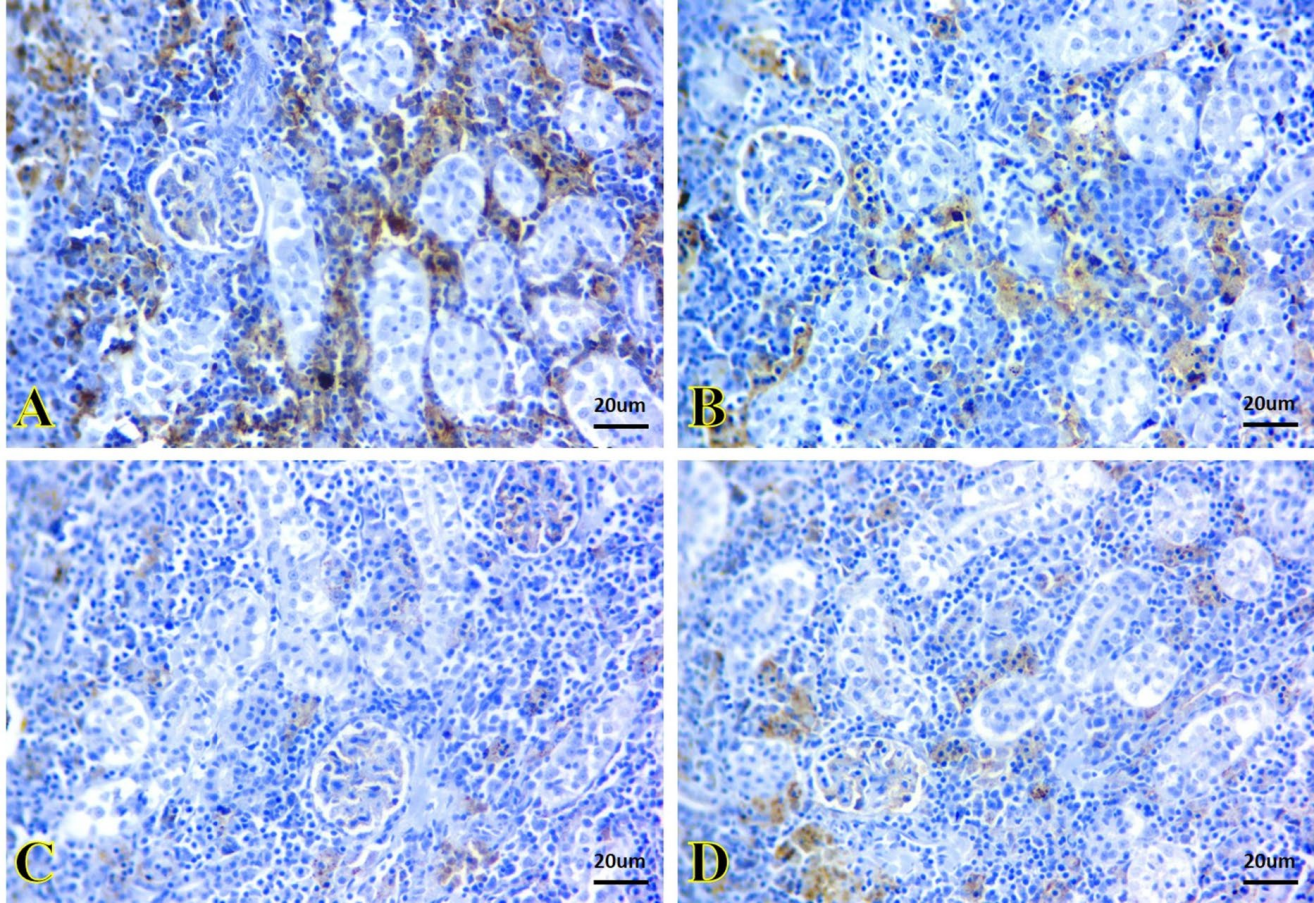
Representative photomicrographs of immunostained kidney sections for BCL-2 immunoreactivity. **A**, **B** The kidney of the control and SiNP groups exhibiting strong immunoreactivity that appeared as brown granules inside the renal tubule cells. **C** The kidney of the *A. veronii* group exhibiting a very weak immunoreactivity. **D** The kidney of the *A. veronii +* SiNP group exhibiting moderate immunoreactivity. Scale bar 20 μm

**Fig. 7:**
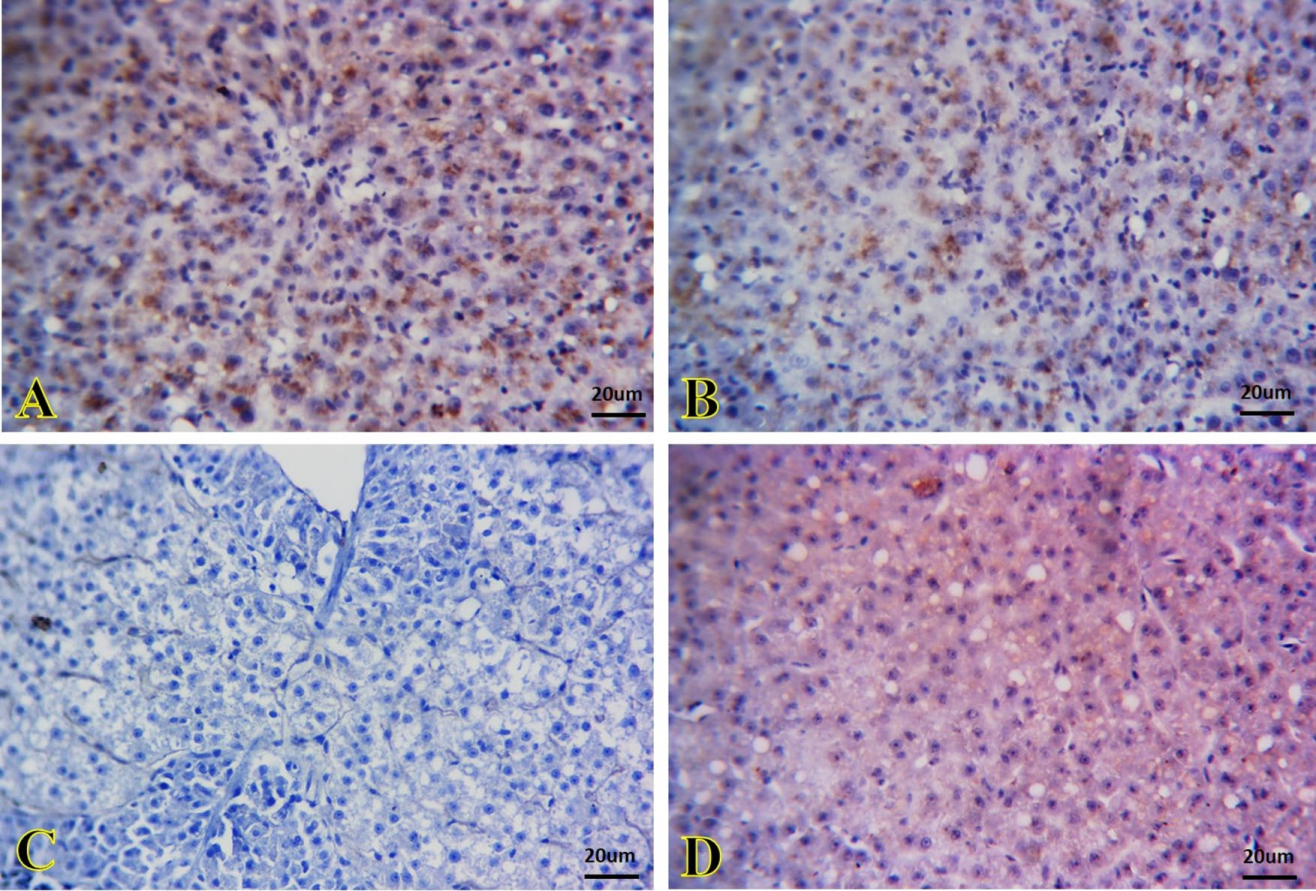
Representative photomicrographs of immunostained liver sections for BCL-2 immunoreactivity. **A**, **B** The liver of the control and SiNPs groups exhibiting strong immunoreactivity that appeared as brown granules inside the hepatic cells. **C** The liver of the *A. veronii* group exhibiting negative immunoreactivity. **D** The liver of the *A. veronii +* SiNP group exhibiting weak immunoreactivity. Scale bar 20 μm

The kidney and liver tissues of the control and SiNPs groups showed potent BCL-2 immunoreactivity that appeared as brown granules inside the renal tubules (Fig. 6A and B) and hepatic cells (Fig. 7A and B), respectively. *A. veronii* infection caused a very weak BCL-2 immunoreactivity in the kidney tissue (Fig. 6C) and a negative immunoreactivity in the liver (Fig. 7C). A moderate immunoreactivity was noted in the kidney of *A. veronii +* SiNP group (Fig. 6D). In contrast, the immunoreactivity was weak in the liver (Fig. 7D).

**Fig. 8:**
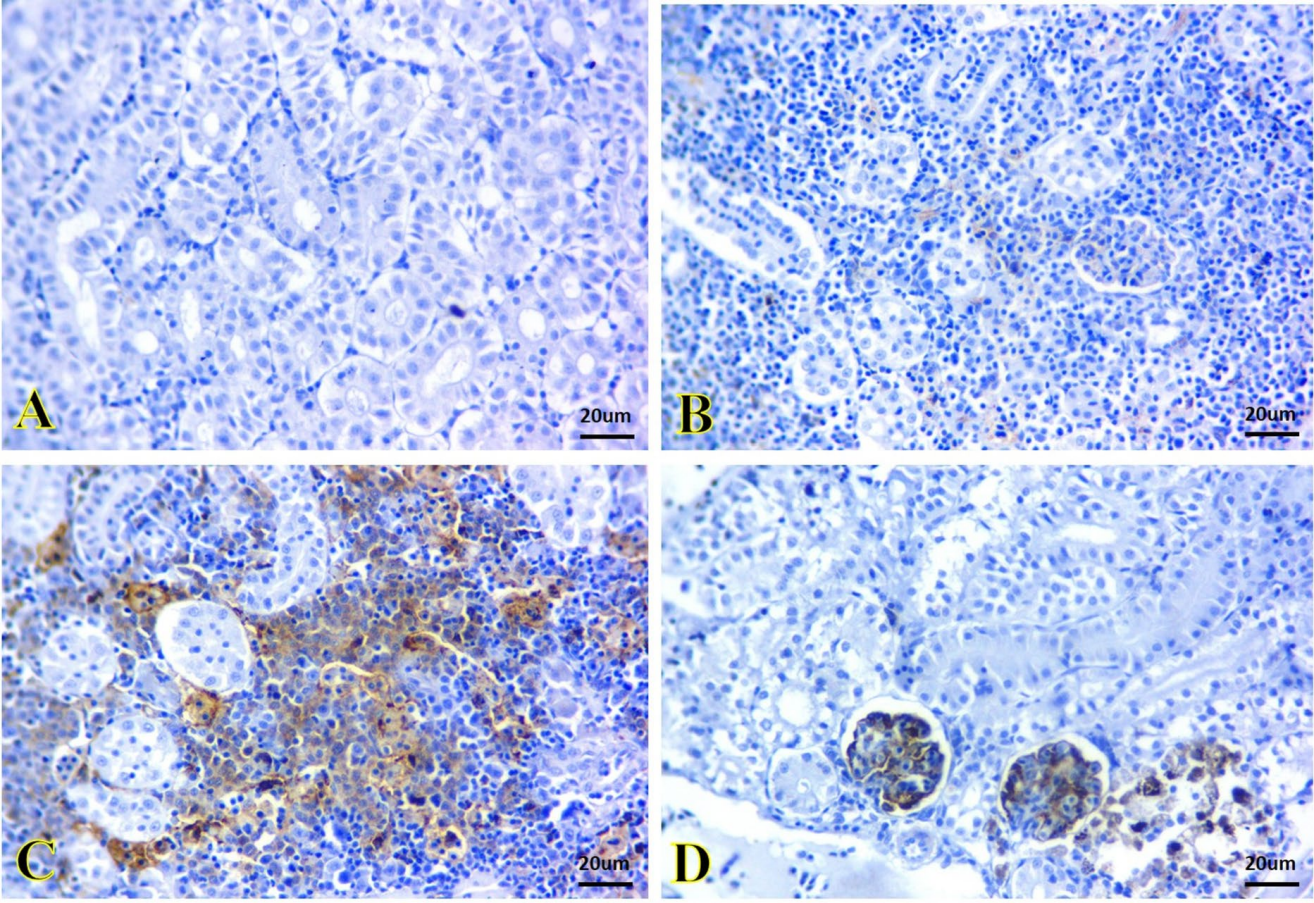
Representative photomicrographs of immunostained kidney sections for caspase-3 immunoreactivity. **A**, **B** The kidney of the control and SiNP groups exhibiting negative immunoreactivity. **C** The kidney of the *A. veronii* group exhibiting strong immunoreactivity that appeared as brown granules inside the cells of the renal tubules. **D** The kidney of the *A. veronii +* SiNP group exhibiting mild immunoreactivity. Scale bar 20 μm

**Fig. 9:**
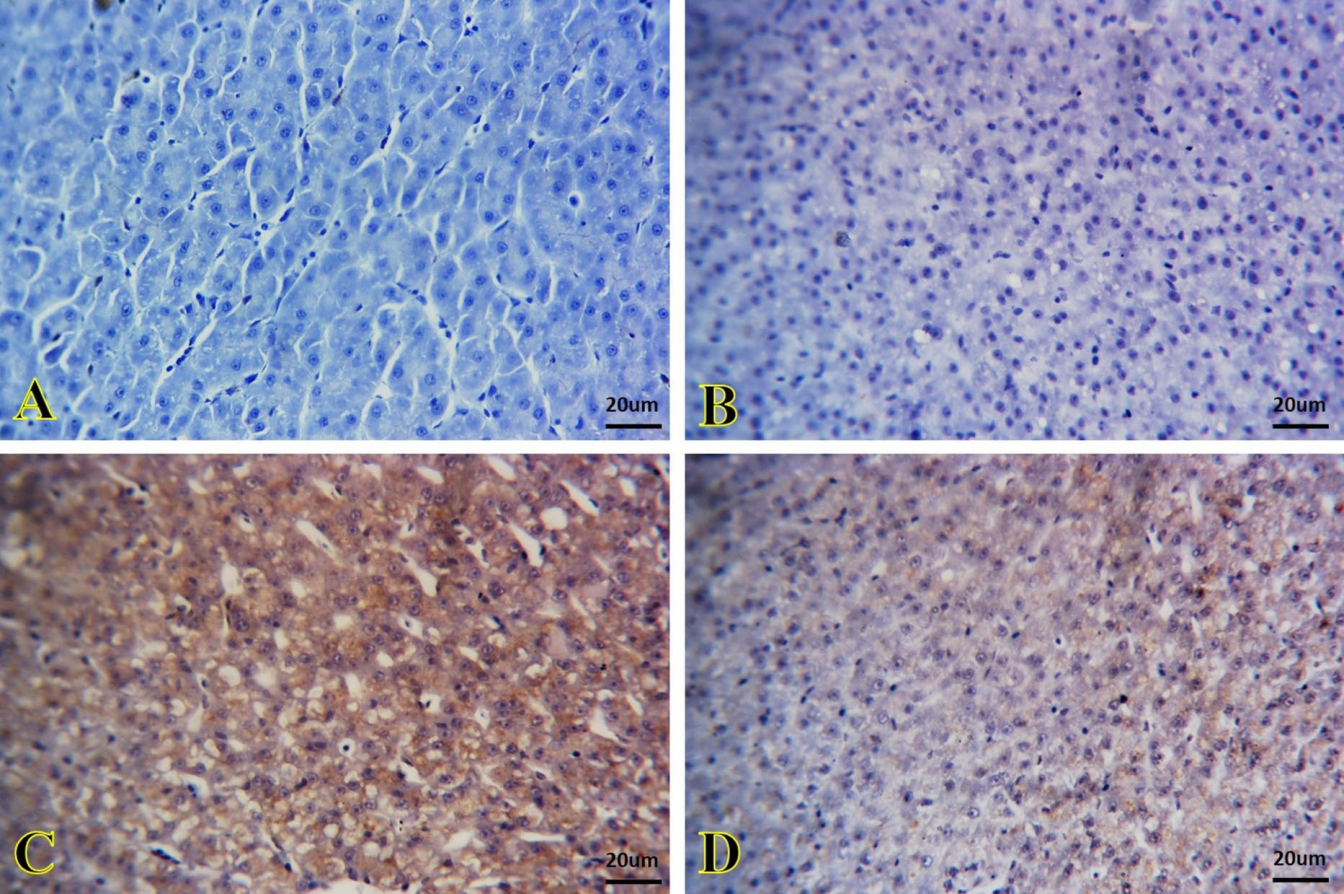
Representative photomicrographs of immunostained liver sections for caspase-3 immunoreactivity. **A**, **B** The liver of the control and SiNPs groups exhibiting negative immunoreactivity. **C** The liver of the *A. veronii* group exhibiting strong immunoreactivity that appeared as brown granules inside the hepatic cells. **D** The liver of the *A. veronii +* SiNP group exhibiting mild immunoreactivity. Scale bar 20 μm

Figures 8 and 9 show a negative immunoreactivity of caspase-3 in the kidney and liver tissues of the control and SiNP groups (Fig. 8A and B, and Fig. 9A and B, respectively). A strong caspase-3 immunoreactivity caused by *A. veronii* infection was observed that appeared as brown granules inside the renal tubules cells (Fig. 8C) and hepatic cells (Fig. 9C). Contrarily, the tissues of the kidney (Fig. 8D) and liver (Fig. 9D) in the *A. veronii +* SiNP group were exhibited mild immunoreactivity.

## Discussion

Due to the huge production in the aquaculture industry, many pathogenic infectious diseases arise that threaten fish life, specifically bacterial infections (Rajme-Manzur et al. 2021). Nowadays, there is extensive use of nanomaterials in the aquaculture sector. Nanoparticles have greatly succeeded in broad use as dietary supplements, water treatment, drug delivery, and disease control (Elabd et al. 2022). Until now, a few reports have addressed the influence of SiNPs against bacterium in African catfish. Therefore, the present work aimed to investigate the potential role of the aqueous addition of SiNPs for mitigating immune-antioxidant suppression, hepato-renal dysfunction, and histopathological and immunohistochemical alterations of African catfish experimentally infected with *A. veronii*.

In assessing ethological changes, the present study clarified that the infection with *A. veronii* induced different behavioral alterations, including spiral movement, loss of equilibrium, and laterality, as well as various clinical signs. It is assumed that the virulence genes of *A. veronii* plus its toxic products are responsible for its pathogenicity, which negatively affects the health and performance of fish, resulting in these abnormalities. As reported by Youssef et al. (2023), *A. veronii* has alt, fla, lipase, aerolysin, and act genes, identifying them as the primary cause of its pathogenicity. Another document verified virulence factors for *A. veronii*, including outer membrane proteins, proteases, toxins, secretory enzymes, and hemolytic and cytotoxic activities (Chen et al. 2019). Likewise, a recent study by Said et al. (2023) found that the infection of Nile tilapia in *A. veronii* resulted in equilibrium loss, hemorrhagic spots, loss of the scales, and fin rot.

Contrarily, the aqueous addition of SiNPs (20 mg/L) to the *A. veronii* challenged group regenerated these alterations and enhanced the fish behavior. It is suggested that the nano-sized SiNPs can easily penetrate cells, resulting in a direct immunomodulatory effect on the fish immune system via elevating values of immune and immunohistochemical biomarkers, as confirmed in our study, which in turn enhanced the health status of fish and, as a consequence, modulated the fish behavior.

The head kidney (anterior kidney) is a powerful hematopoietic organ in fish, while the other part of the kidney (posterior kidney) is responsible for excretion (Bates et al. 2018). The liver is the main organ that plays a pivotal role in the binding, storage, and detoxification (Mahboub and Shaheen 2021). The hepatic enzymes are crucial indicators that reflect the liver’s health status by boosting the host’s antioxidant status against pathogens. Urea and creatinine indicate kidney function (Giri et al. 2018). The current study demonstrated a dysfunction in the hepato-renal organs represented by elevation in renal biomarkers and liver enzymes upon exposure to *A. veronii* plus histopathological alterations in liver and kidney tissues. Such alterations induced by *A. veronii* could be dominated by the release of this bacterium to some virulence factors, including lipopolysaccharide (LPS), proteins, and extracellular factors hemolysins, flagella, lipases (Lip), and proteases (Ser) as previously mentioned by Hossain and Heo (2021). It is opined that these virulence factors could produce oxidative damage in the hepato-renal tissues and reflect noticeable increases in the ALP, ALT, urea, and creatinine levels, as well as histopathological alterations. Concurrently, Adhikary et al. (2023) reported severe histopathological changes in the liver, kidneys, and gills of the Indian major carp (*Cirrhinus mrigala*) post-exposure to *A. veronii* infection. Likely, Said et al. (2023) revealed that *A. veronii* negatively interfered with hepato-renal functions, indicated by elevated levels of ALT, ALP, creatinine, and urea in Nile tilapia as well as clear histopathological lesions in kidneys and liver tissues.

Conversely, exposure to SiNPs in *A. veronii*-challenged fish mitigated the histopathological alterations, regenerated the tissue picture, and improved hepato-renal function biomarkers, implying their protective role as antioxidants. SiNPs could probably lessen the *A. veronii*–induced ROS by penetrating smoothly through renal and hepatic cells, reflecting antioxidant protection. These findings were supported by Ravelo-Nieto et al. (2023), who declared that SiNPs are safe, perfect penetrating agent that can easily pass through biological membranes without influencing the viability of cells. A similar report by Mahboub et al. (2022b) found that SiNPs can alleviate the histopathological changes induced by heavy metal toxicity in *C. gariepinus*.

TP and GLO are active indicators for stimulating the humoral immune response in fish (Castro and Tafalla 2015). LYZ and complement-Cs are components of the non-specific immune system that fish mainly rely on as a primary defense mechanism after bacterial challenge (Giri et al. 2018). The current study revealed that *A. veronii* suppressed the immune response by lessening levels of immune biomarkers. It could be dominated by the pathogenicity of *A. veronii* and secretion of enterotoxins, which depress the capacity of phagocytes to exert their activity on engulfing bacterium and, accordingly, suppress the immune system as documented by Arslan and Küçüksari (2015). In line with Said et al. (2023), *A. veronii* suppressed the immune response of Nile tilapia by inducing a clear reduction in nitric oxide and LYZ activity. Furthermore, Reyes-Becerril et al. (2015) mentioned that *A. veronii* is a pathogenic species to the Pacific red snapper (*Lutjanus peru*) by inducing a state of immune depression.

Herein, we report the immuno-stimulatory effect for SiNPs via augmenting LYZ, C3, and TP levels. It is opined that SiNPs suppress bacterial activity and minimize the dissemination of *A. veronii* through their promising role in enhancing the immune response. The mechanism of action of SiNPs on innate immunity was recently described by Ganesan et al. (2022), which represented the activating of macrophages and T and B cells and enhancing the release of immunoglobulins. Concurrent with a new report, Abdel Rahman et al. (2023a) verified the immunomodulatory role of SiNPs through elevating levels of nitric oxide and immunoglobulin plus up-regulation of the immune-associated genes as interleukins (*IL-8* and *IL-1β*) in *C. gariepinus*. Also, Mahboub et al. (2022b) reported that the aqueous exposure of *C. gariepinus* to SiNPs up-regulated the cytokines *IL-1β* and *IL-8*, which play a key role in regulating the immune response.

GPx is a crucial enzyme responsible for ROS detoxifying and protecting the living organism from oxidative stress (Malandrakis et al. 2014). TAC indicates oxidative damage or increased vulnerability to oxidative stress (Young 2001). MDA is one of the most substantial products of oxidation and is mainly monitored as a marker of oxidative stress (Mendes et al. 2009; Mahboub et al. 2022a). Herein, we report the dangerous impact of *A. veronii* on the antioxidant defense mechanism via altering oxidative/antioxidant biomarkers (elevating MDA and decreasing GPx and TAC). It is assumed that the oxidative stress produced by *A. veronii* is because it produces extracellular enzymes that suppress the antioxidant system. These findings were supported by Yang et al. (2020) and Zhu et al. (2022), who clarified that the oxidative stress produced by *A. veronii* is because of the elevated levels of ROS-generating cytotoxicity. Our study underlines the promising influence of the aqueous addition of SiNPs on antagonizing oxidative damage induced by *A. veronii* via modulating the biomarkers mentioned above, which confirms the potential role of SiNPs as a potent antioxidant. Likewise, a recent document by Abdel Rahman et al. (2023a) mentioned the occurrence of oxidative damage following exposure of *C. gariepinus* to *A. veronii* manifested by a reduction in the catalase, superoxide dismutase, and reduced glutathione content and modulation in these biomarkers in the exposed group to SiNPs. The promising influence of SiNPs could be returned to their effect on sustained cell viability because their nano-scale size enables them to penetrate cells and exert their direct effect against the bacterium. In the same manner, Abdel Rahman et al. (2023b) reported the antioxidant efficacy of magnetite nanoparticles in eliminating oxidative damage induced by *Aeromonas sobria* in *C. gariepinus*.

Apoptosis represents a programmed cell death essential to tissue homeostasis by removing the damaged cells (Raducka-Jaszul et al. 2020). It is monitored by various cellular signaling mechanisms (Edinger and Thompson 2004) and biochemical pathways, which induce cellular alterations involving shrinkage, fragmentation of the nucleus, condensation of chromatin, and fragmentation of DNA, and eventually cause the death of cells (Elmore 2007). Caspase-3 is an important apoptotic marker stimulated when apoptosis is commenced (Wong 2011). Intrinsic apoptosis represents an interaction of the BCL-2 and its membranes (Banjara et al. 2020). Herein, we report a strong apoptosis that appeared as a disturbance of BCL-2 and an upregulation of caspase-3 immuno-historeaction in the kidney and liver tissue of the *A. veronii*-challenged group. This finding was supported by previous studies that linked pathogen apoptosis, cytotoxicity, and DNA damage to the expression of related genes that contribute to hosting adherence, colonization, and occurrence of infection (Hoel et al. 2017; Srivastava et al. 2017). Another report supported our findings and clarified that *A. veronii* up-regulated many inflammatory genes and down-regulated 52 protein-coding genes influencing chromatin regulation and numerous minute nuclear RNAs, plus 55 cytoplasmic tRNAs. Such impacts induced apoptosis via intrinsic pathways (Lee and Lio 2023).

Contrarily, adding SiNPs generated the BCL-2 immunoreactivity and produced weak apoptosis as a down-regulation of the caspase-3 in the *A. veronii*-infected group. This could be an outcome of the protective impact of the nano-sized SiNPs that can easily penetrate cells and protect tissue against the bacterium, resulting in an elevation in the resistance of cells against apoptosis via mitigating apoptotic signaling induced by *A. veronii* reflecting a potent BCL-2 immunoreactivity. Also, the protecting effect of SiNPs against apoptosis could be returned to the reduction of ROS generation, which, in turn, SiNPs can protect cells, as recently documented by Kretowski et al. (2021).

## Conclusion

Building on the outcomes, SiNPs are a new, successful tool to avoid the drawbacks of *A. veronii* bacterial infection in *C. gariepinus*. The aqueous SiNPs (20 mg/L) have a potent antibacterial agent that lessens behavioral changes and improves health status. Moreover, SiNPs noticeably boost the hepato-renal function, immune-antioxidant capacity, and histological parameters after exposure to the *A. veronii* challenge. Further studies are needed to investigate other antimicrobial roles of SiNPs and assess their efficacy on other fish species.

## Data Availability

All data generated or analyzed during this study are included in this article.
